# Machine learning-inspired similarity measure to forecast M&A from patent data

**DOI:** 10.1371/journal.pone.0341010

**Published:** 2026-02-06

**Authors:** Giambattista Albora, Matteo Straccamore, Andrea Zaccaria

**Affiliations:** 1 Centro Ricerche Enrico Fermi, Piazza del Viminale, Rome, Italy; 2 Sony CSL - Rome, Joint Initiative CREF-SONY, Piazza del Viminale, Rome, Italy; 3 Istituto dei Sistemi Complessi (ISC) - CNR, UoS Sapienza, P.le A. Moro, Rome, Italy; Institute of Economics (Scuola Superiore Sant’Anna)/RFF-CMCC European Institute on Economics and the Environment, ITALY

## Abstract

Defining and finalizing Mergers and Acquisitions (M&A) requires complex human skills, which makes it very hard to automatically find the best partner or predict which firms will make a deal. In this work, we propose the MASS algorithm, which adapts a patent-based measure of similarity between companies to forecast M&A deals. MASS is based on an extreme simplification of tree-based machine learning algorithms and naturally incorporates intuitive criteria for deals; as such, it is fully interpretable and explainable. By applying MASS to the Zephyr and Crunchbase datasets, we show that it outperforms a more “black box” graph convolutional network algorithm. The latter, however, turns out to be the most effective algorithm when considering companies with disjoint patenting activities. This study provides a simple and powerful tool to model and predict M&A deals between companies active in patenting, offering valuable insights to managers and practitioners for informed decision-making.

## 1 Introduction

In today’s rapidly evolving landscape of technological advancements, companies face the constant challenge of staying at the forefront of innovation. While internal research and development efforts play a significant role, they may not always be sufficient in terms of time and costs to keep up with the swiftly changing technological environment. As a result, many firms seek to expand their technological horizons by engaging in Mergers and Acquisitions (M&A) [1]. Such operations are used extensively as a financial instrument by firms of any region and size and constitute a business that, only in 2019, has almost reached 4 trillion dollars (https://imaa-institute.org). Such strategic moves allow companies to tap into the technological capabilities (here proxied by patenting activity) of their target entities, leverage their patents, and potentially venture into new markets. The choice of the possible best target for a deal is made in a complex, evolving landscape of partners and competitors, involving a huge effort in terms of time and human capabilities. In this paper, we propose an automatized, machine learning-inspired approach to quantify the closeness between two firms in terms of their patenting activities, and we test this and other measures in an out-of-sample forecast exercise. Equipped with this tool, decision-makers can assess to what extent to exploit a technology sector a firm already masters or explore new innovation possibilities. In order to build a quantitative measure of the similarity between companies, we draw inspiration from the Economic Complexity framework [2]. In particular, our investigation centers on the concept of “Relatedness” [3–5], the idea that the co-location of economic activities traces an overlap in the capabilities they need. In the present work, we reverse this perspective and we measure the similarity between two firms using the technological sectors found in their patents. Our similarity metric allows us to compare and contrast the patent portfolios of acquiring and target companies, enabling a deeper understanding of the technological dynamics at play in these strategic transactions.

Similarity metrics, such as cosine similarity, are the key to constructing collaborative filtering [6], which is a widely employed technique in recommender systems and link prediction exercises. Recently, in the field of unweighted bipartite networks, it has been introduced a novel metric known as Sapling Similarity [7]. This metric has demonstrated superior performance in link prediction and recommendation tasks compared to existing metrics in the literature. In this study, we have modified the Sapling Similarity to predict M&A events, introducing the MASS approach. First, MASS correctly considers weighted bipartite networks, which is the context of our firm-technology network. Second, similarity measures usually assume symmetry, meaning that the probability of firm *f*_1_ acquiring firm *f*_2_ is the same as that of firm *f*_2_ acquiring firm *f*_1_. However, this assumption does not hold in the real world, and MASS considers that it is more likely that a large, established firm acquires a small startup than the other way around. Finally, we included a preference of acquirer firms for rare technologies [8]. The simplicity of the mathematical expression of MASS makes its output (the likelihood of M&A events between two firms) fully interpretable and explainable. Our findings reveal that this approach yields a significant enhancement in our ability to understand and make predictions on future M&As with respect to other methods, including black-box machine learning such as decision tree-based algorithms [9,10] and graph convolutional networks [11].

Furthermore, we have delved deeper into our study by considering the distinctions among firms belonging to different sectors. Certain sectors, such as the pharmaceutical industry, tend to generate patents more frequently, whereas others, like the financial sector, exhibit less frequent patenting activity [12–14]. Notably, when firms possess a low degree in the firm-technology bipartite network, it is common to observe M&A transactions between two firms that have zero co-occurrences, indicating no shared technological codes. In such instances, all traditional similarity metrics, like Cosine Similarity, but also Sapling Similarity fail to capture any meaningful signals. To deal with this situation we discuss the use of a graph convolutional neural network (LightGCN [15]). Our investigation reveals that this machine learning-based approach can deal with events between firms with zero co-occurrences, as it discerns signals that simpler similarity metrics are incapable of detecting.

While in this paper we focus our attention on patenting activity, we point out that, in general, M&A deals are not completely driven by technological reasons. As pointed out in [16–18], financial, cultural, and geographical motivations may coexist. The paper is organized as follows. In Sect 2 we review the related literature both in M&A studies and in the Economic Complexity field. In Sect 3 we briefly discuss our objectives. Sect 4 is devoted to the description of our database and methodologies. We present our results about deal prediction in Sect 5. Sect 6 concludes.

## 2 Literature review

### 2.1 Literature review on M&A

Recently, the literature on mergers and acquisitions (M&A) has expanded significantly, exploring various directions. Given the global scale of these trillion-dollar processes, it has been crucial to conduct studies with an economic focus. For instance, research on risks through success and failure cases [19] and assessments of efficiency indicators for acquiring organizations [20] have been fundamental. The study by Ding et al. [21] examines the role of discriminatory protections, highlighting how regulatory environments and protectionist policies can influence the strategies and outcomes of cross-border M&As. Additionally, [22] investigates the impact of the COVID-19 pandemic on global M&A activity. [23] provide significant insights into the dynamics of M&A activities in emerging markets, especially during uncertain times. Through a comprehensive literature review, they explore how mergers and acquisitions can be utilized as strategic tools for achieving business sustainability amidst fluctuating economic conditions.

A prominent concept in M&A research is Absorptive Capacity [24], which refers to the acquiring company’s ability to identify and acquire externally generated knowledge from the target firm. This concept is closely related to the ideas of Relatedness and Similarity in Economic Complexity and emphasizes how the similarity between the acquirer and target companies is crucial for successful integration [25]. This “capacity” can depend on various factors. For example, geographical distance has been found to negatively influence the probability of M&A occurrences [26,27], while similarities in ownership or industrial sector can increase such probabilities [28,29]. Analyzing a large set of acquisitions using a similarity measure introduced by [30,31] establish a statistically significant correlation between M&A occurrence and industry relatedness.

A notable section of M&A studies focuses on the patenting activities of involved firms, centring on technological relatedness. [32] introduce a measure of technological similarity between acquirer and target firms, revealing an inverse parabolic relationship between technological similarity and innovation performance post-acquisition. Many subsequent authors develop different measures of technological relatedness and investigate their links to post-acquisition performance [33–39]. However, results regarding the inverse parabolic behaviour between relatedness and performance are inconclusive due to the lack of a standardized and recognized method for robust performance and relatedness measurements [37,40].

While the majority of M&A literature focuses on correlating relatedness measures with successive performances, [41] highlights the importance of forecasting as a crucial test for the validity of relatedness assessments. Notable forecast exercises involve [42], which uses an ensemble learning algorithm to predict future acquisitions based on relative features between companies and patent data, and [43], where a tree-based machine learning algorithm is trained on a large set of M&A features encompassing financial, geographical, industrial, and patent data of firms for M&A prediction. In [44], the authors use patent documents to derive a technological profile of companies and predict possible deals, while in [45] SEC annual reports are analyzed. Other possibilities include the use of machine learning to determine the key factors to predict an M&A [46] (but see also [47]). Also the effects of social networks have been analyzed [48]. Finally, in [14], the authors develop a method to predict future acquisitions by assuming that companies deal more frequently with technologically related ones. Following this line of research, and in contrast with the pure forecast exercises discussed above, the aim of this paper is to understand which features of the technological activity of firms are important for a deal to actually occur. For this reason, we adopt a measure of technological similarity and we modify it to take into account features such as the respective size of firms (in terms of patenting activity) and technology rarity. Note that a specific comparison of the prediction performances of the above mentioned approaches would require an investigation of the union of the different datasets they use.

In the next section, we will illustrate the methodological framework we adopt to build our measure of technological, size and rarity-aware, similarity.

### 2.2 Literature review on relatedness

Economic Complexity (EC) is a conceptual framework that studies the knowledge intensity of an economy or an industrial sector by using network-based and machine-learning approaches. One line of research stems from the concept of relatedness [3], which measures the similarity between economic activities or the affinity between an economic actor or a geographical area and such activities, for instance exporting a product or patenting in a specific technological field [49,50,62]. Measures such as Product Space [4] and the Taxonomy Network [5] have become essential tools for policymakers and economists to understand and predict economic dynamics [51].

Innovation, usually measured from patent data, serves as a critical driver of economic complexity. The correlation between a country’s patenting activity and its industrial and scientific development has been extensively documented [52]. Moreover, the future trajectory of a firm can be predicted by the economic or technological value of its early patents [53].

For all these reasons, the concept of technology forecasting plays a crucial role in the field of economic complexity applied to innovation [54–58].

On the other side, the concept of ’Relatedness’ in EC literature has been particularly instrumental in understanding the dynamics of mergers and acquisitions (M&A). Studies have leveraged this concept to predict the likelihood of M&A by examining the technological and product similarities between firms [14,25–27,42,43,59], providing a nuanced view of strategic business decisions. Recent advances have applied EC principles to predictive modelling [7,41,60,61]. However, applying EC, and the concept of Relatedness in particular, to M&A prediction is an innovative frontier that remains underexplored. The intersection of EC measures and recommender systems has opened new research avenues. Adapting algorithms from recommender systems, researchers have begun to address the unique challenges of M&A prediction within the EC framework, conceptualizing firms as both the users and items of a recommendation engine. Despite these advancements, significant gaps remain in the literature, particularly concerning the application of EC to M&A predictive modelling using patent data. This review highlights the need for a more granular approach to consider the nuances of firm-level data and the specificities of technological sectors. This is the main objective of this paper.

We conclude this section by stressing the need for scientific validation to confirm or falsify the proposed assessments of relatedness. Many different (or similar) measures are available in the economic complexity field, and even small variations can produce very different outputs [40]. As a consequence, an out-of-sample forecasting scheme has been introduced [41] to compare relatedness assessments by using the assumption that economic actors will, on average, engage more frequently in more affine, or related, activities. The results point out the need to use tree-based machine learning methods to quantify relatedness [62]. Sapling Similarity [7] extremely simplifies these approaches, making them fully interpretable and explainable, and keeping the accuracy of machine learning forecasts. In this paper, we will follow the same approach, by suitably modifying Sapling Similarity for M&A predictions and by verifying the goodness of our approach by checking the out-of-sample forecasting performance of this in comparison with other possible measures. This empirical verification is key to validating the ability of our measure to capture significant economic features.

## 3 Objectives and contribution

In this study, we address the problem of forecasting mergers and acquisitions deals by analyzing the patenting activities of firms by using network-based and machine-learning approaches borrowed from the Economic Complexity and the recommender system literature. Our purpose is to shed light on the underlying patterns and strategic motivations behind such transactions also contributing to the ongoing discussion on the intricate relationships between technological capabilities, diversification strategies, and corporate performance.

The main contributions of this paper are as follows:

1. Through the exploration of the relationship between patent data and M&A, we advance our understanding of the dynamics that shape the innovative landscape of modern businesses.
2. We introduce the MASS algorithm, which represents a generalization of the Sapling Similarity that can take continuous values as input, and takes into account the different sizes of the technological portfolios of the acquirer and target company and the likely seek of rare technologies.
3. We use the MASS algorithm to forecast new deals, finding that it outperforms other approaches in both predicting new acquirer-target couples and future targets given an acquirer.
4. In cases of limited or not overlapping patenting activities, where MASS is hardly applicable, graph convolutional neural networks represent the best option to assess the likelihood of a deal. Note that at least a minimum amount of patenting activity must still be present.
5. The majority of recommendations typically involve connecting nodes from different layers of the bipartite input network. In other words, in recommender systems usually the input is a bipartite user-item network, and items are recommended to users [63]. Here, by comparing the respective portfolios of items (i.e., the technology codes relative to the patenting activities), we recommend users to users (i.e., we predict which firm will make a deal with which firm), adopting a monopartite setting.

## 4 Data and methodology

### 4.1 Data

The study utilized a combination of four distinct databases to gather relevant data. Information regarding patent classifications and technological areas was sourced from the PATSTAT database (www.epo.org/searching-for-patents/business/patstat). The AMADEUS database (https://login.bvdinfo.com/R0/AmadeusNeo) facilitated the linkage of patents with their respective companies. Additionally, details regarding M&A were collected using the Zephyr (https://www.bvdinfo.com/it-it/le-nostre-soluzioni/dati/greenfield-investment-and-ma/zephyr) and Crunchbase (www.crunchbase.com) databases. This section will elaborate on the dataset preprocessing techniques applied and detail the construction of the database that facilitated the development of similarity indices and forecasting models.

#### 4.1.1 Patent data.

In this investigation, patent and technology data were derived from the Worldwide Patent Statistical Database (PATSTAT, https://www.epo.org/searching-for-patents/business/patstat.html), a resource maintained by the European Patent Office (EPO). PATSTAT serves as a repository, systematically gathering data from diverse patent authorities at regional and national scales. PATSTAT also aggregates patent applications filed at different times or in different countries that refer to the same invention into what are known as patent families (in the present work, we used the earliest filing year to identify the year of the family). To ensure that the patents considered have undergone similar scrutiny and represent high-value, internationally protected inventions, the selected patents are from so-called triadic patent families, i.e., those that have been filed not only with the EPO but also with the Japanese Patent Office (JPO) and the U.S. Patent Office (USPTO). This approach is widely adopted in the literature [64].

The core utility of this database in our research lies in its adoption of the International Patent Classification (IPC) system, an organized hierarchy sanctioned and routinely revised by the World International Patent Organization (WIPO). This system provides a uniform method to organize patents by technological content through a hierarchical structure of codes, ranging from more than 70,000 detailed categories at the lowest level to eight expansive sections at the uppermost. For instance, under this system, “A” commonly represents “Human Needs,” and “E” signifies “Fixed construction”. More specifically, “A01” covers sectors like “Agriculture; Hunting,” and “E04” is associated with “Building”. This analysis intentionally omits categories marked as “99” and subclasses labeled “Z,” which are allocated for atypical technologies that are not categorized under standard classifications, thus are not analyzed herein. Further exploration and details on how this dataset is applied in practice can be reviewed in the work of [65], where the methodology and applications are discussed extensively. In this work, we adopt a 7-digit disaggregated approach to capture the intricate combinations of technological fields. Robustness checks confirm that our results hold across different levels of aggregation.

#### 4.1.2 Firm data.

The AMADEUS database (https://login.bvdinfo.com/R0/amadeusneo) provided the company-related data for this research, documenting details on over 20 million companies which primarily covers European companies, with non-European firms represented through their European subsidiaries. Managed by Bureau van Dijk Electronic Publishing (BvD), this resource is notable for its detailed records on the financial, operational, and fiscal aspects of corporations. One of the key features of the AMADEUS database under BvD’s stewardship is the synchronization of patent identifiers with those maintained by the European Patent Office, enhancing the compatibility with the PATSTAT database for comprehensive joint analyses [65]. While the AMADEUS database is comprehensive for larger firms, it is recognized that its coverage of smaller firms, especially those employing fewer than 20 individuals, is not as extensive [66]. Nevertheless, this limitation is of minimal consequence to the goals of our current research.

Finally, it is important to point out that only firms with active patent activity were considered for this study. This selection, though not fully representative of all M&A participants, ensures that our analysis focuses on firms where technological innovation is most salient. Nevertheless, as shown in S1 Fig (Supporting information), the sectoral distribution of M&A participants within our patenting sample closely matches that of all patenting firms, supporting the representativeness of our dataset within the innovation-active population.

#### 4.1.3 Dataset from Crunchbase and Zephyr for M&A.

The investigation drew on merger and acquisition (M&A) data collected from two separate sources: Zephyr and Crunchbase. Zephyr (https://www.bvdinfo.com/en-us/our-products/data/greenfield-investment-and-ma/zephyr), operated by Bureau van Dijk Electronic Publishing (BvD), archives comprehensive data on global M&A activities, Initial Public Offerings (IPOs), and ventures in Private Equity and Venture Capital, along with speculative market activities. This study specifically tapped into Zephyr’s data on the biopharmaceutical sector, which includes around 4000 recorded transactions involving more than 3700 companies from 1997 through 2016. Conversely, Crunchbase (https://www.crunchbase.com) serves as a broad repository initially conceptualized for tracking start-up ecosystems. It provides detailed insights into both public and private entities, encompassing acquisitions, mergers, and broader investment activities on a global scale. In comparison to Zephyr, Crunchbase boasts a more expansive database, chronicling over 100000 acquisition events since 1922 and detailing the activities of more than one million companies.

#### 4.1.4 Dataset creation.

The construction of our dataset began with the AMADEUS-Patstat database, which creates a bipartite network of companies linked to technology codes from their patents, as elucidated in the work of [65]. Companies are identified by BVDID, which correlates with their patent technology codes by weight, indicating patent share per technology. Incorporating the Zephyr dataset, we directly mapped 430 companies to their technological profiles from an initial pool of 3167 M&A-involved entities. For the Crunchbase dataset, a name-cleaning algorithm was employed to match company names to their BVDIDs, culminating in 12017 companies being linked to appropriate technological portfolios out of 28137 candidates. Where multiple BVDIDs emerged, typically for multinational entities with various subsidiaries, we consolidated the corresponding technological portfolios. The M&A analysis, confined to the period between 2002 and 2012 and to companies with patenting activity from 2000, yielded a sample of 1279 M&A events across 1974 companies. Crunchbase’s proprietary industrial sector taxonomy, featuring 744 categories and 43 category groups, was refined into 13 aggregated sectors. This reclassification allowed us to distinctly categorize 8069 firms, which represents approximately 70% of the companies aligned with their technological portfolios. For our analyses, we selected a subset of the M&A dataset that exclusively includes companies with a singular sector designation based on our refined classification. This subset comprises 8737 companies, with 913 participating in 547 M&A transactions. While this restriction excludes highly diversified firms, it ensures a cleaner and more interpretable benchmark by reducing the ambiguity in sector classification. Importantly, robustness tests performed in previous work [14], where negative samples were drawn from the full set of companies, including diversified ones, showed that while absolute performance metrics may vary, the relative ranking of predictive methods remains stable. This confirms that the use of firms with clearly defined sector labels does not bias the evaluation in favor of our method.

The temporal aspect of the dataset is encapsulated in 13 yearly adjacency matrices, $M^{y}$, spanning 2000 to 2012. These matrices chart the relationships between 8737 companies and 7132 technologies, with each element $M_{ft}^{y}$ signifying the affiliation of a firm *f* with a technology *t* for a specific year *y*. The matrices are constructed by assigning a uniform weight to each patent, distributed among all pertinent firm-technology pairs and aggregated annually. This method recognizes that patents may cover several technologies and are rarely filed by multiple firms. We extend the analysis by considering cumulative matrices $M^{Y}$, each summarizing the technological involvement of firms from 2000 to year *Y*, to reflect a firm’s evolving innovation profile. These cumulative matrices underpin our predictive models, which hypothesize that the similarity in technological portfolios between companies can forecast potential M&A activities in year *Y*.

The resultant dataset, employed in the subsequent analyses of this paper, features 8737 companies, of which 913 were involved in 547 M&A deals. The companies were selected based on the availability of a unique industrial sector from the Crunchbase data, enhancing the precision of our predictive modeling.

### 4.2 Methods

In this section, we describe the various similarity metrics used to assess the similarity of companies from their patents. The starting point is the matrix $M^{Y}$ whose element *M*_*ft*_ is the number of patents firms *f* submitted in the technological sector *t* during year *Y*. In the following, we will omit the year specification to lighten the notation. This matrix is the representation of a bipartite firm-technology network, and the matrix elements quantify the weights of such a network. Given an acquirer firm A and a target firm T, we want to compute *B*_*AT*_, the likelihood of firm A acquiring firm T; different methods will provide different estimations of *B*_*AT*_. We will then use these assessments to predict M&A deals.

#### 4.2.1 Notation.

In the following, we will define the matrix Λ as the result of the scalar product between **M** and its transpose, $M\cdot M^{T}$. This matrix is square, and each row represents a possible acquirer company, while each column represents a possible target company. The matrix element $\Lambda_{AT}$ represents the scalar product between the row vectors A and T of the **M** matrix. Note that if **M** is binary, $\Lambda_{AT}$ is equal to the number of co-occurrences $CO_{AT}=\sum_{\lambda }M_{A\lambda }M_{T\lambda }$, i.e. the number of technologies firms *A* and *T* share.

We will denote the row vector of Λ corresponding to firm A as $\Lambda_{\left(A\right)}$ and the column vector of Λ corresponding to firm T as $\Lambda^{\left(T\right)}$. Finally, we will utilize the notation $max(\Lambda_{\left(A\right)})$ to denote the maximum element of the $\Lambda_{\left(A\right)}$ vector, $max(\Lambda^{\left(T\right)})$ to denote the maximum element of the $\Lambda^{\left(T\right)}$ vector and max(Λ) to denote the maximum element of the matrix Λ.

#### 4.2.2 Cosine similarity.

The first measure we introduce is cosine similarity, also known as Jaffe similarity in the context of M&A, as it was introduced in 1986 by Jaffe to quantify the productivity of manufacturing R&D [67]. Since then, in the work of Valentini and Dawson [68], the cosine similarity measure was applied in the M&A context. More recently, [14] conducted a comparative analysis of various methodologies, including machine learning algorithms, for estimating the likelihood of a deal. They concluded that the most effective approach involves assessing the similarity in technological portfolios between the two companies, utilizing cosine similarity as the metric. In our notation, the equation for cosine similarity reads

$$B_{AT}^{Jaf}=\frac{\sum_{\lambda }M_{A\lambda }M_{T\lambda }}{\sqrt{\sum_{\lambda }M_{A\lambda }^{2}}\sqrt{\sum_{\lambda }M_{T\lambda }^{2}}}=\frac{\Lambda_{AT}}{\sqrt{\sum_{\lambda }M_{A\lambda }^{2}}\sqrt{\sum_{\lambda }M_{T\lambda }^{2}}}$$ (1)

#### 4.2.3 Sapling similarity for unweighted bipartite networks.

**Fig 1:**
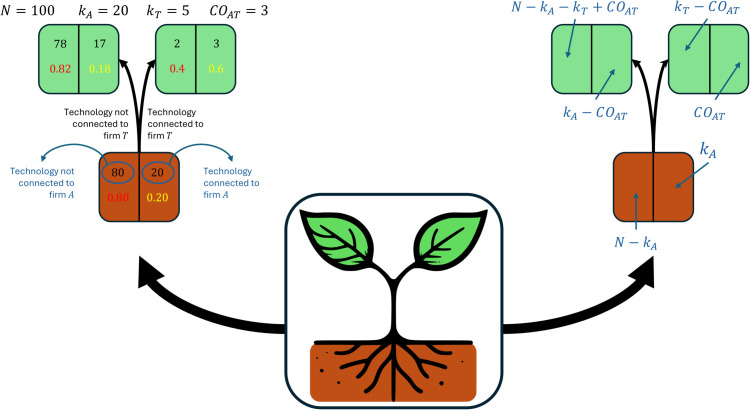
On the left figure, an example of a decision sapling illustrating the relationship between two firms, A and T, in a scenario featuring 100 technologies. The node at the bottom indicates the percentage of technologies that are (right) and are not (left) connected to firm A; in this figure, A is connected to 20% of technologies, as denoted by the green value. The upper nodes show how these percentages change when considering only technologies connected to Firm T (right node) or only those not connected to Firm T (left node). On the right figure, we show the value of each box as a function of the number of co-occurrences CO, the degrees k, and the total number of technologies *N*.

Albora et al. [7] have recently introduced a new metric of similarity between nodes in unweighted bipartite networks: the Sapling Similarity. In this paper, the bipartite network under study will be the one that connects companies to the technology sector mentioned in their patents. The idea behind the Sapling Similarity metric is to extract the main ingredients of tree-based machine learning models, since they outperform other approaches in Relatedness estimations (see [62]), to allow full interpretability and explainability while preserving the prediction performance. Sapling Similarity is an indicator ranging from –1 to 1 that reflects how similar the technological portfolios of two firms are. Given firms A and T, while cosine similarity evaluates the angle between their technological portfolio vectors, Sapling Similarity examines how the information that T is active in a generic technological class C alters our prior probability that A is active in C. If this probability increases, then the Sapling Similarity between A and T is positive; if it decreases, then the similarity is negative. In order to visualize how Sapling Similarity works, in Fig 1 we present what is referred to as a ‘decision sapling’ in [7].

On the left, we represent a numerical example in which only *N* = 100 technologies exist and two firms, A and T, are considered. A is connected to (i.e., has patenting activity in) *k*_*A*_ = 20 technologies, and T is connected to *k*_*T*_ = 5 technologies. A and T share *CO*_*AT*_ = 3 technologies. The bottom node indicates the fraction of the technologies that firm A is either connected with or not; notably, firm *A* has established connections with 20% of the technologies, as denoted by the green value. Upper nodes detail the updates of these proportions when the analysis is narrowed to technologies either connected with or not connected with firm *T*. In this example, knowing that a technology is connected with T increases the probability that it is connected to A from 20% to 60%, suggesting a positive similarity between A and T.

To quantify this line of reasoning, we need a measure of the polarization in a node. Given *p*_1_ the fraction of positive samples in a decision tree node, and *p*_0_ the fraction of negative samples, the Gini Impurity of the node [69] is defined as:

$$GI=1-p_{0}^{2}-p_{1}^{2}=2p_{0}p_{1}.$$ (2)

This quantity tells us how much the samples in the node are peaked, or concentrated, towards the positive or the negative case: maximum polarization (only positive or negative samples) implies *GI* = 0 while minimum polarization (equal number of positive and negative samples) means *GI* = 0.5. So if we consider the lower node of the decision sapling, we have *p*_0_ = 0.8, *p*_1_ = 0.2, and *GI*^*low*^ = 0.32. Analogously we find that the Gini Impurity of the upright node is *GI*^*upr*^ = 0.48 and the one of the upleft node is *GI*^*upl*^ = 0.29. The variation of the Gini impurity is defined as:

$$\frac{\Delta GI}{GI^{low}}=\frac{GI^{low}-f^{upl}GI^{upl}-f^{upr}GI^{upr}}{GI^{low}}$$ (3)

where *f^upl^* and *f^upr^* are the fraction of samples that are respectively in the upper left and the upper right node (in the case of the figure, 0.95 and 0.05).

The variation of the Gini Impurity quantifies how much the information that T patents or not patents in a generic technology field *λ* is important to understand whether A patents in *λ*. This is the absolute value of the Sapling Similarity; its sign is positive if $p_{1}^{upr}\geq p_{1}^{low}$ (which means that knowing that T is connected to *λ* increases the probability that also A is connected to it), and negative otherwise.

Using the figure on the right, in which we report the general formulas, we can easily derive an equation for the sapling similarity as a function of the co-occurrences *CO*_*AT*_ (how many technologies firms A and T share), the degrees *k*_*A*_ and *k*_*T*_ (the number of technologies each firm has) and the total number of technologies in the bipartite network *N*:

$$B_{AT}^{sap}=\left\{ \begin{array}{c} 1-f_{AT}\;\text{ if }\frac{CO_{AT}N}{k_{A}k_{T}}\geq 1\\ -1+f_{AT}\;\text{ otherwise } \end{array}\right.$$ (4)

Where:

$$f_{AT}=\frac{CO_{AT}\left(1-\frac{CO_{AT}}{k_{T}}\right)+\left(k_{A}-CO_{AT}\right)\left(1-\frac{k_{A}-CO_{AT}}{N-k_{T}}\right)}{k_{A}\left(1-\frac{k_{A}}{N}\right)}.$$ (5)

#### 4.2.4 Generalization of the sapling similarity for weighted bipartite networks.

In our specific case, the input data is a bipartite network that connects firms to the technology fields, which is weighted; so we need to generalize the Sapling Similarity, which in previous papers has always been used with binary inputs. Our line of reasoning goes as follows. The degree *k*_*F*_ of a firm *F* can be seen as the maximum number of co-occurrences that this firm can have (indeed, it cannot share with another firm a number of technologies larger than the number of its technologies); on the other hand, we can think of N as the maximum number of co-occurrences that two generic firms can have (that is, the number of co-occurrences between two firms that possess all the technologies).

In the case in which the matrix **M** takes continuous values, the number of co-occurrences between the two firms A and T is generalized as the scalar product between the two row-vectors of the matrix **M** that represent the patenting activity of A and T.

$$CO_{AT}\overset{\text{continuous}\;}{\to }M_{A}\cdot M_{T}=\sum_{\lambda }M_{A\lambda }M_{T\lambda }\overset{\text{def}}{=}\Lambda_{AT}$$ (6)

To simplify the following equations we introduce the matrix Λ whose elements are defined in Eq 6. Since the elements of **M** take continuous values without an upper limit, in principle the theoretical maximum value of $\Lambda_{AT}$ is infinite. However, we can take this value from the empirical counterpart: so we consider $\max(\Lambda_{\left(A\right)})$ as the equivalent of *k*_*A*_ and $\max(\Lambda^{\left(T\right)})$ as the equivalent of *k*_*T*_. With the same reasoning, we can say that the continuous equivalent of *N* is max(Λ). So with these changes, the equation of the sapling similarity in the continuous case reads:

$$B_{AT}^{sap}=\left\{ \begin{array}{c} 1-f_{AT}\;\text{ if }\frac{\Lambda_{AT}\max(\Lambda )}{\max(\Lambda_{\left(A\right)})\max(\Lambda^{\left(T\right)})}\geq 1\\ -1+f_{AT}\;\text{ otherwise } \end{array}\right.$$ (7)

Where:

$$f_{AT}=\frac{\Lambda_{AT}\left(1-\frac{\Lambda_{AT}}{max\left(\Lambda^{\left(T\right)}\right)}\right)+\left(\max\left(\Lambda_{\left(A\right)}\right)-\Lambda_{AT}\right)\left(1-\frac{\max\left(\Lambda_{\left(A\right)}\right)-\Lambda_{AT}}{\max\left(\Lambda \right)-\max\left(\Lambda^{\left(T\right)}\right)}\right)}{\max\left(\Lambda_{\left(A\right)}\right)\left(1-\frac{\max\left(\Lambda_{\left(A\right)}\right)}{\max\left(\Lambda \right)}\right)}$$ (8)

#### 4.2.5 The MASS algorithm: Considering firms’ size and technologies’ ubiquity.

The generalization of the Sapling Similarity discussed above allows one to apply it to continuous variables. This is a first step in building our algorithm to forecast M&A deals, as the number of patents companies file in a given technology sector is non-binary.

In order to obtain the MASS measure, which is optimally designed to forecast M&A deals, we introduce three modifications to the Sapling Similarity framework:

- **Continuous inputs:** We generalize the Sapling Similarity to weighted bipartite networks, where the input matrix contains the number of patents rather than binary indicators. This is implemented by replacing co-occurrence counts with scalar products between firms’ patent vectors (Eqs 7 and 8).
- **Firm size asymmetry:** To reflect that larger firms are more likely to acquire smaller ones (and not vice versa), we introduce an asymmetry based on firm size, approximated by the norm of the firm’s patent vector. This leads to a rescaling of the similarity score:
  $$B_{AT}^{sap(1)}=\frac{\sqrt{\sum_{\lambda }M_{A\lambda }^{2}}}{\sqrt{\sum_{\lambda }M_{T\lambda }^{2}}}B_{AT}^{sap}$$ (9)
- **Technology rarity weighting:** We boost the impact of shared rare technologies by down-weighting common technologies in the similarity computation. This is implemented by modifying the scalar product to penalize technologies with high ubiquity:
  $$\Lambda_{AT}=M_{A}\cdot M_{T}\to {\tilde{\Lambda }}_{AT}\overset{\text{def}}{=}\sum_{\lambda }\frac{M_{A\lambda }M_{T\lambda }}{v_{\lambda }}\;\text{with }v_{\lambda }=\sqrt{\sum_{f}M_{f\lambda }^{2}}.$$ (10)

In the results section, we refer to the term SS as Sapling Similarity, which can be either weighted or unweighted and is utilized without the adjustments (1) and (2) reported above. By SS(1), we denote the inclusion of the modification defined in Eq 9; SS(2) includes Eq 10, and SS(1+2) includes both. The metric we designate as MASS (Mergers and Acquisitions Sapling Similarity) corresponds to the weighted version of SS(1+2).

#### 4.2.6 LightGCN.

In the context of recommendation systems, Graph Convolutional Networks (GCNs) [15,70] have become increasingly popular due to their ability to capture complex relationships within data. Essentially, GCNs work by learning features from graph structures, such as networks of users and products, by considering the connections and the features of neighbouring nodes.

Light Graph Convolutional Network (LightGCN), introduced by [11], is a streamlined variant of the traditional GCN, specifically designed for recommendation systems. LightGCN simplifies the GCN architecture by removing feature transformation and non-linear activation functions. This simplification aims to reduce computational complexity while maintaining, or even enhancing, the performance in recommendation tasks.

LightGCN operates directly on the user-item interaction graph. It effectively learns user and item embeddings by aggregating features from neighboring nodes, capturing both direct and indirect interactions within the graph. This approach allows LightGCN to efficiently and accurately model the preferences and behaviors of users, leading to improved recommendation quality.

In this study, we will use LightGCN to predict M&A and we will compare its performance with cosine Similarity (that outperforms various machine learning approaches, see the work of [14], Sapling Similarity, and the newly introduced MASS.

## 5 Experiments

### 5.1 Pair, target, and acquirer prediction

In this paper, we aim to compare the effectiveness of various methods in predicting future M&A deals between companies. We estimate the probability of the possible M&As occurring between companies in a given year Y using only past data; so the prediction scores are recalculated in each year. To assess the quality of the methodologies, we examine their ability to predict M&As between companies. In practice, this translates into three distinct binary classification exercises:

1. Pair Prediction: For this exercise, the 547 pairs of companies that undergo M&A are labeled as positive events, while negative labels are assigned to randomly generated pairs of companies. For each M&A that occurs in year Y, 200 negative pairs are generated, ensuring that each pair is unique and not among the 547 actual M&A pairs. The best-performing model is the one that accurately distinguishes the true M&A pairs from the randomly generated ones.
2. Target Prediction: Here, for each of the 547 actual acquirers, 200 negative targets are generated ensuring that these do not coincide with the real targets and that there are no repetitions. The optimal model is identified as the one that can effectively differentiate the actual target of the single acquirers from those randomly generated.
3. Acquirer Prediction: Similar to the target prediction, this exercise involves generating 200 negative acquirers for each of the 547 true targets. The model’s task is to identify the true acquirer of each target.

In all three types of experiments, for every single M&A deal, 200 negative company pairs are generated, resulting in a class imbalance of 1:200 in the binary classification exercise. To quantify the performance of the models in these three types of experiments, we use standard performance indicators for binary classification [71,72]. For pair prediction, we employ the following three indicators:

- Best F1 score (best F1) [41,62,73]: This score is computed by finding the threshold that maximizes the F1 score [74], that is defined as the harmonic mean of precision and recall;
- Area under the Precision-Recall Curve (AUC PR) [75,76]; the area under the curve on the precision-recall plane. This area is derived by varying the threshold that determines the score above which predictions are classified as positive.
- Precision at 500 (prec@500); To compute this metric, we evaluate the top 500 scoring elements. prec@500 measures the proportion of these top 500 elements that are true positives (accurately predicted positively) out of the total 500 elements examined.

For target and acquirer prediction, we utilize:

- Best F1 score (best F1), as for pair prediction;
- Hit Ratio at 5 (HR 5) [77]: it measures the proportion of times that the relevant item (the true acquirer in the acquirer prediction exercise and the true target in the target prediction exercise) appears in the list of the top 5 recommendations;
- Mean Average Precision (mAP): the mean of the average precision [78] across all acquirers (target prediction exercise) or targets (acquirer prediction exercise);

Each experiment is repeated 20 times, with negative M&As being regenerated each time. The final score is an arithmetic mean of these 20 repetitions.

### 5.2 Results

**Fig 2:**
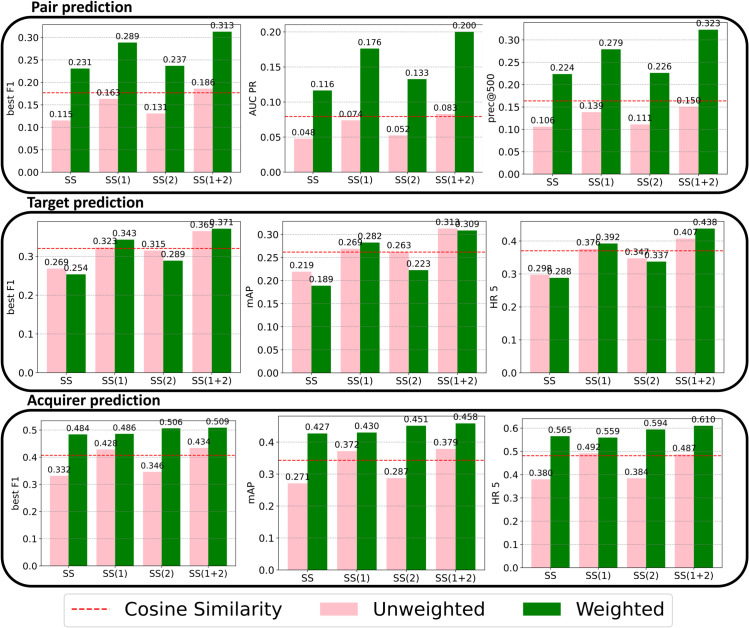
Performance of the various Sapling Similarity variants in predicting M&A deals in the three cases: pair, target, and acquirer prediction. The variants, reported on the x-axis, are described in the Methods section. We report in pink the case in which the input network binary, and in green, the weighted case. The MASS algorithm corresponds to the green SS(1+2) variant. The red line shows the performance of cosine similarity measured using the weighted network. All improvements enhance the prediction performances, MASS being the outperforming algorithm.

The prediction performances of the different methods are compared in Fig 2, which shows nine bar plots arranged in a 3x3 grid. Each row corresponds to a different prediction exercise: pair prediction, target prediction, and acquirer prediction, respectively. Within each row, three distinct performance indicators are utilized for evaluation: In order Best F1 Score, Area Under Precision Recall Curve, and Precision at 500 for pair prediction; Best F1 Score, Mean Average Precision, and Hit Ratio at 5 for both target and acquirer predictions. The bar plots feature eight bars, grouped in pairs, with pink bars representing predictions made using an unweighted network and green bars for those made using a weighted network. Each pair of bars corresponds to a variant of sapling similarity, incorporating modifications (1) and (2), introduced in the methods section, aimed at refining the prediction accuracy for this particular case study. The current state of the art is cosine similarity measured using the weighted network (red dashed line).

The modifications introduced to the base equation of Sapling Similarity consistently yield improvements. This enhancement aligns with the rationale behind their introduction: smaller target companies are generally the focus of acquisitions, addressed by modification (1), and the attractiveness of a small company possessing a rare technology, as considered in modification (2).

Generally, predictions utilizing a weighted network outperform those based on an unweighted network, validating the effectiveness of extending Sapling Similarity to incorporate the case of weighted networks. However, in target prediction scenarios, the distinction between weighted and unweighted networks is minimal. This suggests that for large companies acquiring smaller ones, the mere possession of a technology by the target is significant, irrespective of whether it is the target’s main technology or not. In contrast, understanding the importance of technologies to the acquiring company is crucial for accurately predicting the acquirer.

The MASS approach, incorporating strategic modifications to the already well-performing Sapling Similarity, consistently outperforms the previous state-of-the-art, that is cosine similarity (the red dashed line in the plots), which previous studies shown to perform better than a number of other approaches [14]. This superiority across all metrics and prediction exercises underlines the method’s robustness and its capacity to set a new benchmark in the field of M&A prediction.

Given that target prediction and acquirer prediction utilize the same performance indicators, a direct comparison reveals that acquirer prediction exercises generally achieve higher performance metrics. This indicates that predicting the acquirer in a M&A scenario is more straightforward than identifying the target.

### 5.3 Acquisitions with low co-occurrences

In the previous section, our investigation has shown the robustness of the MASS approach as the state-of-the-art approach for forecasting M&As between companies from their patenting activity. However, a pertinent inquiry arises regarding the limitations of this method.

Indeed, not all companies exhibit the same propensity for patenting, leading to a significant number of firms with very few technologies - or links in the bipartite network language. This variance can largely be attributed to diverse patenting policies across different sectors. Specifically, certain industries are naturally inclined to generate fewer patents than others. Consequently, some sectors tend to have limited patenting activity, leading to a higher likelihood of M&A deals occurring between firms with zero co-occurrences. Despite its prowess, methods like MASS struggle to predict these instances due to their reliance on shared technologies for similarity calculation.

Among the 547 M&A instances in our dataset, 123 involve pairs of companies with no direct technology overlap, highlighting a substantial subset where Sapling Similarity for M&A’s predictive power is limited. This is where machine learning comes to help.

**Fig 3:**
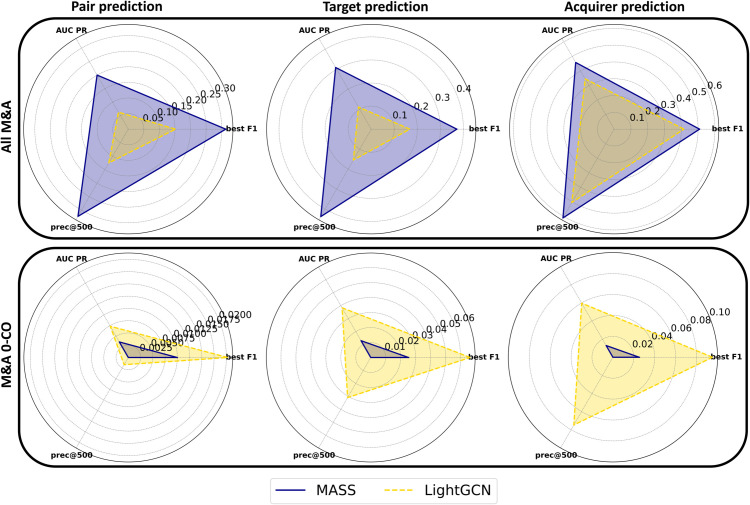
Comparative analysis of MASS and LightGCN performances across different M&A prediction scenarios. The top row of radar plots presents the performance metrics for both methods when the test is conducted on the entire dataset of 547 M&As. Here, MASS outperforms. The bottom row depicts the same exercises, but the test focuses exclusively on the subset of 123 M&As between companies with zero co-occurrences (0-CO). In this case, LightGCN is able to capture the hidden, higher-order similarities between companies.

In Fig 3, we compare the performances of MASS and LightGCN across two different testing scenarios: the entire dataset of 547 M&As (above). and the subset of 123 M&As between companies with zero co-occurrences (below). The radar plots schematically represent the prediction ability of the two approaches in the three different exercises, the largest area being relative to a higher performance. We note that in generating random pairs of companies for negative test cases, care was taken to ensure that none of the pairs matched the 547 actual M&A instances, even when only 123 M&As are considered. Within these plots, the vertices correspond to the three performance metrics utilized in our evaluation. The resulting shapes formed by connecting these vertices thus visually encapsulate the comparative performance of MASS and LightGCN across these metrics.

The graphical representation clearly illustrates that while MASS outperforms LightGCN across the board when considering the full dataset, its supremacy diminishes in scenarios involving companies with no technology overlap, i.e. with zero co-occurrences (0-CO). In these cases, LightGCN emerges as the superior predictive tool. This shift can be attributed to LightGCN’s ability to leverage the broader structure of the bipartite network, capturing latent similarities between companies beyond direct technological co-occurrences. By effectively utilizing this comprehensive network information, LightGCN demonstrates a pronounced advantage in predicting M&As among companies that, on the surface, share no common technological ground.

## 6 Conclusions

This study provides significant insights into the dynamics of Mergers and Acquisitions (M&A), utilizing patent data and the Economic Complexity framework, and in particular neural networks and machine learning-inspired algorithms. Starting from the bipartite firm-technology network, we estimate the likelihood of an M&A deal occurring between two patenting firms. The recent study of [14], using the same data, demonstrated that cosine similarity between firms is a good estimator of this probability, outperforming other similarity measures and even machine learning approaches. In this study, we have outperformed this result by introducing the M&A Sapling Similarity (MASS) approach. Moving from the Sapling Similarity, a metric recently introduced in the work of [7], we generalize it to take into account weighted bipartite networks; furthermore, we add two modifications that account respectively for the fact that acquirer firms are usually large and target firms are small and that when counting co-occurrences between two firms, those between rare technologies should weigh more in estimating the probability of a M&A.

The results from three different prediction exercises (pair prediction, target prediction, and acquirer prediction) show that our method represents the state-of-the-art in estimating the probability of an M&A occurring between two firms. Furthermore, this study also investigates the case of working with firms that produce few patents. In such cases, it is common to encounter M&As between firms with zero technologies in common. In these instances, methods like Cosine Similarity and MASS have limited predictive power since they hardly detect a similarity signal between the two firms. In this scenario, we show that LightGCN, a graph convolutional network introduced in the work of [11] outperforms other approaches.

We point out that M&A deals are not exclusively driven by technological similarity and often also financial, cultural, and geographical considerations are taken into account [43]. In this paper, these variables are not considered, and we focus our prediction exercise on the comparison among the effects of patenting similarity, technology scarcity, and size asymmetry - once again, measured in terms of patents only; we acknowledge that considering other measures of size such as the number of employees or operating revenue could lead to different results. We also acknowledge that accurately matching patent data to firms is a potential limitation of our work. Although we have mitigated this issue by integrating multiple data sources and employing robust name-cleaning and consolidation techniques, some matching inaccuracies may still persist. Future research aimed at refining these matching methodologies could further enhance the predictive accuracy of our approach. Moreover, Large Language Models could be used to find matching patterns between patents’ texts [79]; this effect could be considered for future work.

This paper advances theoretical knowledge on the intersection of Economic Complexity, technological innovation, and M&A activities; beyond this peculiar application, indeed, the MASS algorithm - or other suitable modifications - represents a first attempt to adapt recommending systems approaches, which are usually relative to bipartite networks - to a monopartite case study. Finally, our work provides actionable insights for practitioners involved in strategic planning and corporate finance.

## Supporting information

S1 File Additional methodological details and robustness checks related to the analysis.
(PDF)
