# Supplementary material for: Machine learning-inspired similarity measure to forecast M&A from patent data
Source: PLoS One. 2026 Feb 6;21(2):e0341010. doi: 10.1371/journal.pone.0341010 (PMC12880743; doi:10.1371/journal.pone.0341010)
Supplement: S1 File — Additional methodological details and robustness checks related to the analysis. (PDF) [file pone.0341010.s001.pdf]

# Supplementary Information

## Machine learning-inspired similarity measure to forecast M&A from patent data

Giambattista Albora, Matteo Straccamore and Andrea Zaccaria

# Contents

**1 Sectoral representativeness and predictive analysis 3**

**2 4-digits results 3**

# 1 Sectoral representativeness and predictive analysis

Table 1 reports the sectoral distribution of all patenting firms and those involved in M&A transactions. The table directly compares absolute counts and relative shares across aggregated industry categories. The distribution of M&A firms closely mirrors that of all patenting firms, confirming the representativeness of our sample and the absence of significant sectoral biases. This descriptive evidence supports the general validity of our findings and indicates that the observed patterns are not driven by specific industrial domains.

Table 1: **Sectoral composition of firms involved in M&A.** Counts and relative shares of patenting firms (Total) and firms involved in M&A transactions (M&A) across aggregated industry categories. The similar proportions across columns confirm the representativeness of the M&A sample.

| **Industry** | **Total (n)** | **M&A (n)** | **Total (%)** | **M&A (%)** |
|---|---|---|---|---|
| Software and Gaming | 942 | 196 | 11.7% | 14.4% |
| Information and Comunication Techonology | 988 | 184 | 12.2% | 13.5% |
| Energy and Transport | 1001 | 151 | 12.4% | 11.1% |
| Medical Research | 830 | 144 | 10.3% | 10.6% |
| Research | 598 | 117 | 7.4% | 8.6% |
| Health Care | 606 | 109 | 7.5% | 8.0% |
| Hardware & Software | 450 | 107 | 5.6% | 7.8% |
| Manufacturing and Goods | 653 | 103 | 8.1% | 7.6% |
| Marketing | 552 | 71 | 6.8% | 5.2% |
| Services and Security | 391 | 63 | 4.8% | 4.6% |
| Economy and Finance | 372 | 50 | 4.6% | 3.7% |
| Community Services | 447 | 44 | 5.5% | 3.2% |
| Clothing and Food | 239 | 25 | 3.0% | 1.8% |

Building on this descriptive evidence (Table 1), we further assess predictive performance under sectoral constraints. Figure 1 reports the corresponding results for both acquirer and target prediction tasks, evaluated with a class imbalance of 150, ensuring that negative samples are drawn from the same sector as the true acquirer or target (indicated by the "Sector" label in the plots). This setup avoids distortions when sectors contain fewer firms and ensures that negatives are realistic competitors.

As expected, introducing sectoral constraints reduces absolute performance scores across all models, reflecting the increased difficulty of the task. However, the relative ordering of models remains stable across both the sector-constrained and unconstrained settings. This robustness indicates that our predictive results are not driven by cross-sectoral differences, but instead capture genuine differences in model effectiveness.

# 2 4-digits results

To verify the robustness of our findings with respect to the level of technological code aggregation, we replicate the entire prediction pipeline by compressing all patent classifications to the 4-digit level of the International Patent Classification (IPC) system. Figure 2 shows the results of this robustness check. The structure of the plot mirrors that of Figure 2 in the main text, reporting the performance of different variants of Sapling Similarity, including the MASS algorithm, across the three prediction tasks: pair, target, and acquirer prediction. As in the main analysis, the MASS algorithm (green SS(1+2)) outperforms cosine similarity (red dashed line) and all other Sapling variants in all settings. While absolute performance scores are slightly lower due to the reduced information in the 4-digit

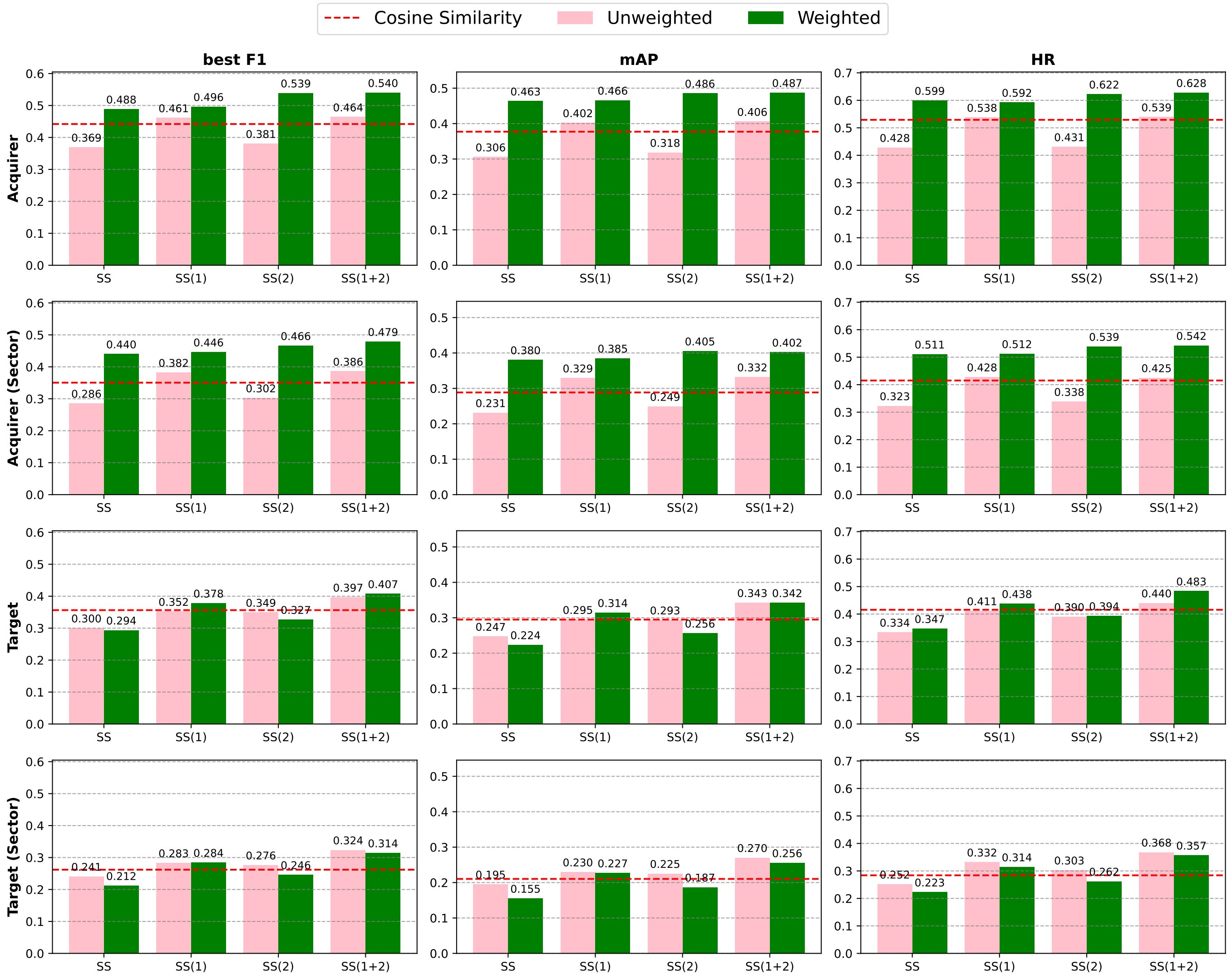


Figure 1: **Predictive performance across sectors.** Comparative results for acquirer and target prediction tasks with and without sectoral constraints (150 negative samples per case). Each subplot reports best F1, mAP, and HR metrics for different model variants, contrasting unweighted and weighted implementations. The dashed line indicates the baseline Cosine Similarity. While sectoral restrictions lower absolute scores, the ranking of models remains consistent, demonstrating the robustness of predictive validity across industrial domains.

codes compared to the full-length classifications, the relative ranking of the methods remains stable. This confirms that the predictive advantage of MASS is not dependent on a particular level of technological detail and that the improvements introduced-weighted input, firm size asymmetry, and rarity correction-continue to provide consistent benefits. This robustness test supports the general applicability of our approach in contexts where highly detailed patent data may not be available or where a more aggregated representation is preferable for interpretability or data constraints. To further explore the relationship between the two levels of aggregation, we compare the pairwise similarities computed with Sapling Similarity using 6-digit and 4-digit IPC codes. The scatter plot in Figure 3 shows a clear positive relationship between the two measures, with correlations of Pearson = 0.89 and Spearman = 0.85, indicating that the overall structure of technological proximities is well preserved despite the aggregation. The few deviations from the diagonal correspond to cases where distinct 6-digit subclasses collapse into broader 4-digit groups, thus reducing granularity but not altering the main topology of the similarity space.

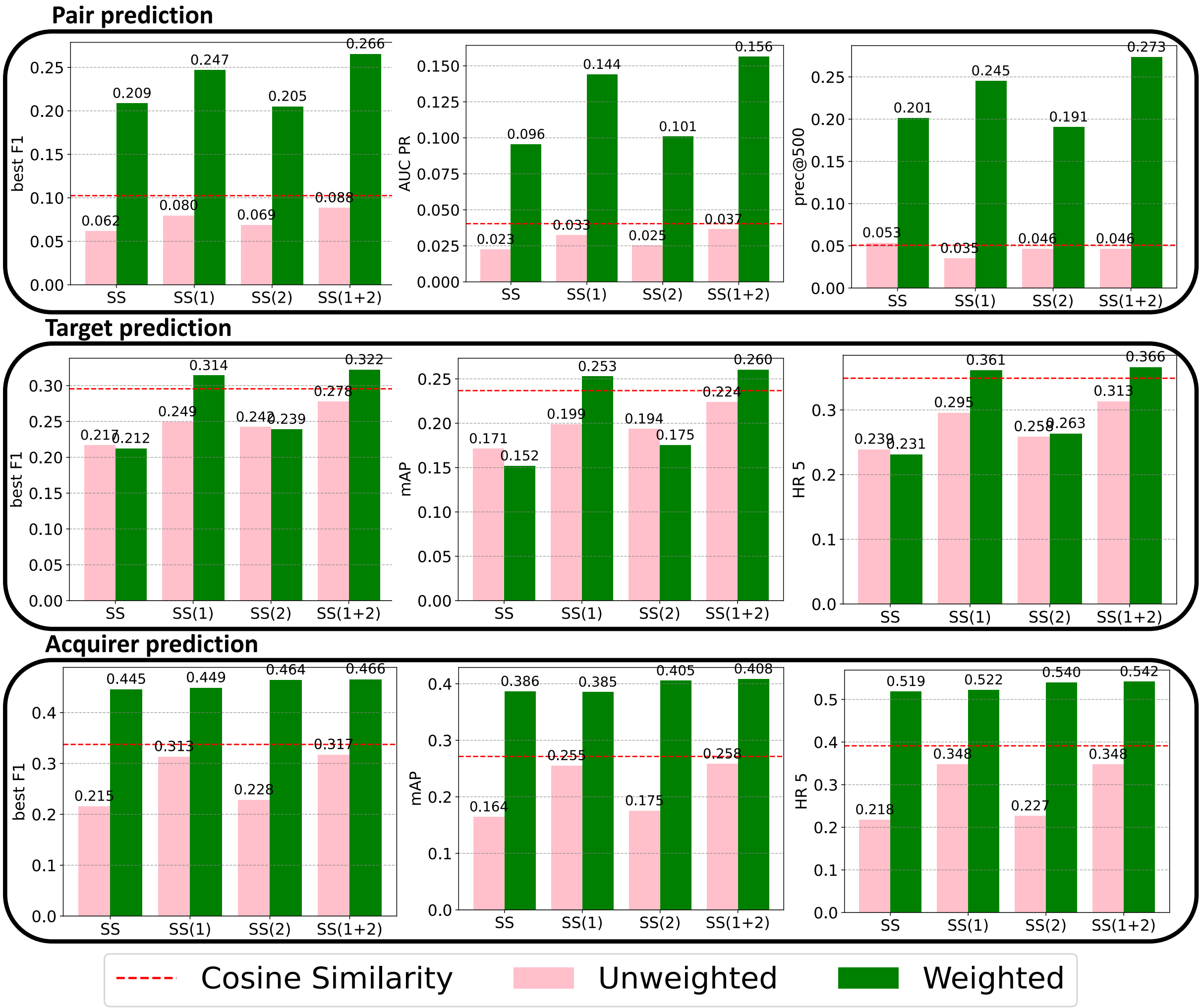


Figure 2: Performance of the various Sapling Similarity variants in predicting M&A deals using 4-digit technological codes. The evaluation covers the three prediction tasks: pair, target, and acquirer prediction. All variants, labeled on the x-axis, are explained in the Methods section of the main text. Pink bars refer to binary input networks, while green bars represent weighted networks. The MASS algorithm corresponds to the green SS(1+2) variant. The red dashed line indicates the performance of cosine similarity computed on the weighted network. Results confirm that MASS consistently outperforms other methods also at this aggregation level.

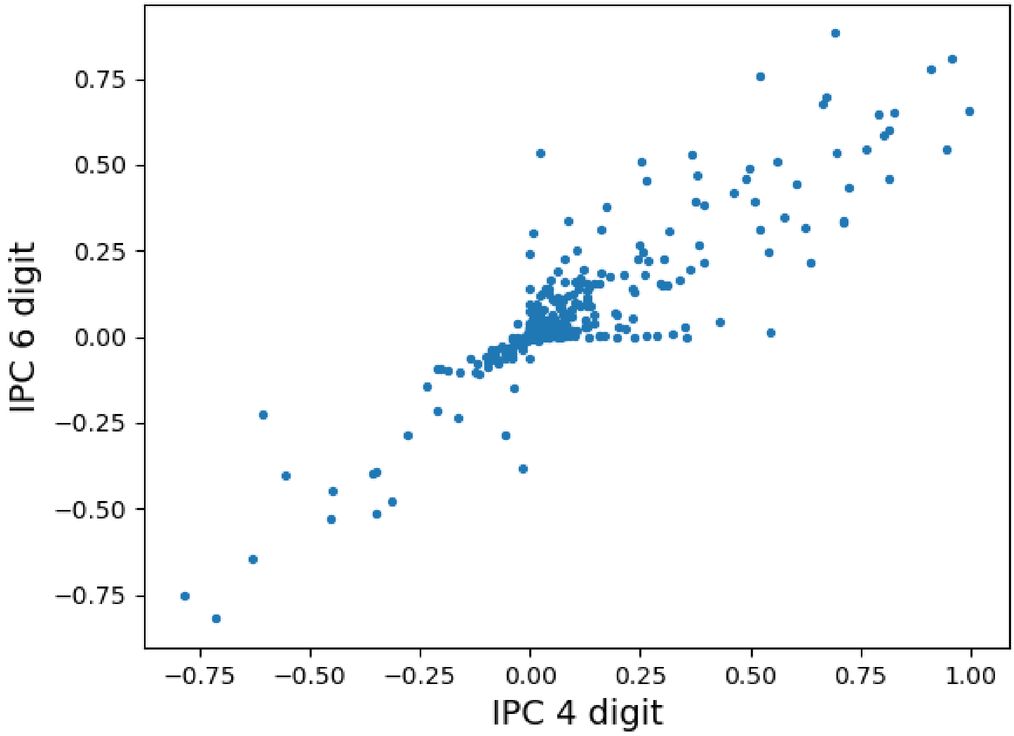


Figure 3: Comparison between Sapling Similarity values computed with 6-digit and 4-digit IPC codes. Each point represents a pair of technological classes. The strong alignment along the diagonal (Pearson = 0.89, Spearman = 0.85) shows that the aggregation from 6 to 4 digits preserves the global similarity structure while slightly reducing the fine-grained variability.
